# Acupuncture and electroacupuncture for stroke: A protocol for overview of systematic review and meta-analysis

**DOI:** 10.1097/MD.0000000000028496

**Published:** 2022-01-07

**Authors:** Tae-Young Choi, Lin Ang, Ji Hee Jun, Hye Won Lee, Jong-Min Yun, JiHee Kim, Byung Soon Moon, Min Cheol Joo, Myeong Soo Lee

**Affiliations:** a Korean Medicine Science Research Division, Korea Institute of Oriental Medicine, Daejeon, South Korea; b Korean Convergence Medicine, University of Science and Technology, Daejeon, South Korea; c Korean Medicine Convergence Research Division, Korea Institute of Oriental Medicine, Daejeon, South Korea; d Department of Korean Internal Medicine, College of Korean Medicine, Wonkwang University, Iksan, South Korea; e Department of Rehabilitation Medicine and Institute of Wonkwang Medical Science, Wonkwang University School of Medicine, Iksan, South Korea.

**Keywords:** acupuncture, meta-analysis, overview, protocol, stroke, systematic review

## Abstract

**Background:**

The aim of this study was to undertake a systematic overview of meta-analyses and published systematic reviews to identify whether and when acupunctureand electroacupuncture are deemed efficacious treatment options for stroke and stroke-related disorders.

**Methods:**

Four databases, namely, PubMed, AMED, EMBASE, and the Cochrane Library will be searched from their inception. Two reviewers will independently perform study selection, data extraction, and assessment. This will be followed by an assessment of the methodological and report quality using the Assessment of Multiple Systematic Reviews-2 tool. Finally, the study will entail the assessment of evidence quality by employing the Grading of Recommendations Assessment, Development, and Evaluation system.

**Results:**

This overview is expected to provide data on using acupuncture for stroke and stroke-related disorders on the basis of the included systematic reviews’ qualitative and quantitative syntheses.

**Conclusion:**

This overview will assess the benefits as well as hazards of acupuncture for stroke, subsequently providing patients and practitioners with useful information and have implications for future studies on the topic.

**Trial registration number:**

Reviewregistry1263

## 1 Introduction

Classified as hemorrhagic or ischemic, stroke is associated with the following symptoms: speech disorder, sudden consciousness disorder, and motor disorder caused by circulatory system challenges like rupture as well as the closure of cerebrovascular accidents.^[1]^ Stroke is a global health problem as it is the leading cause of death and disability worldwide.^[2–4]^ The high sociological and demographic costs of stroke assume significance.^[5]^ Recently, the need to take preventive and countermeasures has been increasingly felt due to the increase in the aging population, which is expected to give rise to increased prevalence and mortality caused by stroke.^[4]^ The myriad stroke and post-stroke symptoms include headache, dizziness, visual field disturbances, hemiparesis, dysphagia, dysarthria, and paresthesia. In the absence of a standard treatment method, a conservative line of treatment is almost always the norm. These symptoms end up affecting the patient's quality of life and stymie their rehabilitation process.

The World Health Organization (WHO) recommends acupuncture (AT) as a complementary and alternative strategy to treat stroke and to enhance stroke care. Widely accepted in western countries, AT has been shown to have a positive effect in stroke patients by several clinical trials,^[6–8]^ both as an alternative and complementary medicine for post-stroke rehabilitation and as a preventive strategy capable of inducing cerebral ischemic tolerance, particularly in conjunction with modern electrotherapy.^[9,10]^ The efficacy of AT in enhancing balance function has been demonstrated by clinical trial findings.^[11,12]^ Similarly, improvements have been noted in cognitive impairment,^[8,13]^ and motor function.^[6]^ According to an overview of meta-analysis (MAs) and systematic review (SRs) conducted in 2020, AT can make improvements in cognitive functions and depressive disorder.^[14]^ However, publication bias and the assessment of only some post-stroke symptoms may have affected the study's reliability.

There is ambiguity in the mechanisms underpinning the benefits of AT. For this reason, the efficacy of AT has always been shrouded with significant debate and controversy.

Plenty of SRs have been conducted where AT and electroacupuncture (EA) treatment have been applied to alleviate post-stroke symptoms. While these SRs have indicative positive results, these findings are far from exhaustive. An overview can potentially augment access to evidence dispersed across multiple SRs. Therefore, this study aimed to synthesize evidence in a systematic manner from SRs and MAs to encapsulate the impact of AT/EA on stroke or stroke-related disorders.

## 2 Methods

### 2.1 Study registration

The registry of this protocol was done on Research Registry with reviewregistry1263 being the registration number (https://www.researchregistry.com/browse-the-registry#registryofsystematicreviewsmeta-analyses/registryofsystematicreviewsmeta-analysesdetails/61a9734b68bc05001eafab79/).

### 2.2 Inclusion criteria

#### 2.2.1 Types of studies

This overview will include all Cochrane SRs as well as non-Cochrane SRs of randomized control trials (RCTs), evaluated the efficiency of AT to treat all types of stroke/stroke-related disorders, regardless of whether they combined data within meta-analyses or not. These disorders include post-stroke dysphagia, depression, hiccups, urinary incontinence, hand-shoulder syndrome, and shoulder pain. However, other overviews and narrative reviews will be excluded.

#### 2.2.2 Types of participants

The selection of participants that will be included in this overview is not restricted by gender, race, or age. However, for their inclusion to take place, they are required to be diagnosed with stroke (intracerebral hemorrhage/cerebral infarction) or stroke-related disorders, such as depression, post-stroke dysphagia, urinary incontinence, hiccups, hand-shoulder syndrome, and shoulder pain.

#### 2.2.3 Types of interventions and controls

The intervention is inclusive of AT as manual AT and EA. MAs and SRs with AT-related treatments such as laser AT, point injection, blood-letting, or transcutaneous electrical nerve stimulation (TENS) will be excluded. To include the trial, when AT is provided together with active treatments for the AT group, this active treatment has to be given to control groups. Also to be included in this overview are control treatments with sham AT, western medicine, placebo, and no treating or waiting list. SRs and MAs with other forms of control group will be excluded.

#### 2.2.4 Type of outcome measures

The outcome measures include SRs reported data concerning at least one outcome interest—stroke clinical efficacy and clinical symptoms. Safety outcomes are not an inclusion criterion for SRs despite being assessed as an additional outcome.

### 2.3 Electronic searches

The English databases that will be searched for pertinent MAs and SRs from their inception to December 2021 are as follows: PubMed, Embase, Allied and Complementary Medicine Database (AMED), and the Cochrane Library. The Medical Subject Heading (MeSH) terms comprising the following search terms will be utilized for searching the database: (“stroke” OR “cerebrovascular accident” OR “infarction” OR “ischemic stroke” OR “apoplexy” OR “hemorrhage”) AND (“acupuncture” OR “electro-acupuncture”) AND (“systematic review” OR “meta-analysis”).

### 2.4 Study selection and data extraction

Two reviewers (TYC and LA) will undertake an independent screening of the titles as well as abstracts for the studies (searched ones), undertake the selection of studies, and record their decisions based on predefined criteria. Disagreements in study selection will be resolved by the third reviewer (MSL). The documentation and summarization of study selection will be done on a flow chart compliant with preferred reporting items for systematic reviews and meta-analysis.^[15]^ After a systematic review of inclusion, the data extract includes publication year, author, search date, the number of searches databases, the number of primary studies (total sample size), type of comparator, type of AT, quality assessment tool, outcome measures, overall bias risk, conclusion (quote from the original paper), effect estimates for main outcomes (meta-analysis), and adverse events (refer to Supplementary 1).

### 2.5 Methodological quality

The quality of reporting for each included systematic review will be critically appraised by the Assessment of Multiple Systematic Reviews-2 tool. A validated 16-item instrument will be used to critically appraise systematic reviews assessing critical flow and bias using ratings of “yes,” “partial yes,” or “no.” The rating of overall confidence in systematic reviews’ findings is made in the following 4 categories: “Critically low” (>1 critical flaw with or without non-critical weaknesses), “Low” (one critical flaw with or without non-critical weaknesses), “Moderate” (more than one non-critical weakness), and “High” (no or one non-critical weakness).^[16]^

### 2.6 Certainty of evidence (CoE)

The Grading of Recommendations Assessment, Development, and Evaluation (GRADE) approach will be adopted to evaluate CoE for all outcomes. Two reviewers (TYC and LA) will undertake an independent assessment of the evidence about outcomes; the downgraded/upgraded factors that impact the evidence's quality will be elucidated to ensure that the results are reliable and transparent. The factors pertained to the risk of inconsistency, bias, precision, indirectness, as well as publication bias. The GRADE approach (GRADEpro GDT is as follows: GRADEpro Guideline Development Tool [Software] McMaster University and Evidence Prime, Inc. 2015. Ontario, Canada) categorizes CoE as high, moderate, low, and very low.

### 2.7 Data analysis

Due to information obtained from overlapping RCTs between SRs, a quantitative analysis of the included SRs will not be performed. The included studies’ narrative description will be provided. This will then be followed by a descriptive summarization of literature search results and data extraction results.

### 2.8 Ethics and dissemination

Since the protocol relates to the SR overview, there is no need to seek patient consent and ethical approval. The overview's results will either be presented at relevant conferences or get published in peer-reviewed journals.

## 3 Discussion

This overview is expected to undertake data provision on the utilization of AT/EA for stroke or stroke-related disorders on the basis of the included SRs’ qualitative and quantitative syntheses. Persistent attempts have been made to treat stroke with AT, as a result of which many SRs on the AT's efficacy for treating stroke have been published.^[17–19]^ However, no overview has synthesized the evidence presented in these SRs in a systematic manner. For this reason, an overview is important in that it will assess the hazards and benefits of AT/EA for stroke or stroke-related disorders, also offering useful information for patients and practitioners, while also having ramifications on future studies on this topic.

## Author contributions

**Conceptualization:** Tae-Young Choi, Min Cheol Joo, Myeong Soo Lee.

**Data curation:** Jong-Min Yun, JiHee Kim.

**Formal analysis:** Tae-Young Choi, Ji Hee Jun, Hye Won Lee.

**Funding acquisition:** Min Cheol Joo, Myeong Soo Lee.

**Methodology:** Lin Ang, Myeong Soo Lee.

**Software:** Lin Ang, Hye Won Lee, JiHee Kim.

**Supervision:** Myeong Soo Lee.

**Validation:** Jong-Min Yun, Byung Soon Moon, Myeong Soo Lee.

**Writing – original draft:** Tae-Young Choi, Myeong Soo Lee.

**Writing – review & editing:** Lin Ang, Ji Hee Jun, Hye Won Lee, Jong-Min Yun, JiHee Kim, Byung Soon Moon, Min Cheol Joo.

## Supplementary Material

Supplemental Digital Content
