# Supplementary material for: Acupuncture and electroacupuncture for stroke: A protocol for overview of systematic review and meta-analysis
Source: Medicine (Baltimore). 2022 Jan 7;101(1):e28496. doi: 10.1097/MD.0000000000028496 (PMC8735753; doi:10.1097/MD.0000000000028496)
Supplement: Supplemental Digital Content [file medi-101-e28496-s001.docx]

**Supplementary 1.** Characteristics of included SRs/MAs

| First author (year)  Country | Search date  No. of searches databases  No. of primary studies (Sample size) | Intervention | Comparator | Outcome | Quality assessment tools  Overall risk of bias | Effect estimates for main outcomes (meta-analysis) | Conclusion  (quote from the original paper) | Adverse events |
| --- | --- | --- | --- | --- | --- | --- | --- | --- |
|  |  |  |  |  |  |  |  |  |
|  |  |  |  |  |  |  |  |  |
|  |  |  |  |  |  |  |  |  |
